# Socio-cultural contextual factors that contribute to the uptake of a mobile health intervention to enhance maternal health care in rural Senegal

**DOI:** 10.1186/s12978-019-0800-z

**Published:** 2019-09-12

**Authors:** Margaret E. MacDonald, Gorgui Sene Diallo

**Affiliations:** 1 York University, 4700 Keele Street, Toronto, ON M3J1P3 Canada; 2 Africare – Senegal, Sacré Coeur 3, villa n°, 9994 Dakar, Senegal

**Keywords:** Mobile health (mHealth), Maternal health, Global health, Senegal, Ethnography, Critical medical anthropology, CommCare

## Abstract

**Background:**

Although considerable progress has been made in reducing maternal mortality over the past 25 years in Senegal, the national maternal mortality ratio (MMR), at 315 deaths per 100,000 live births, is still unacceptably high. In recent years a mobile health (mHealth) intervention to enhance maternal health care has been introduced in rural and remote areas of the country. CommCare is an application that runs on cell phones distributed to community health workers known as *matrones* who enroll and track women throughout pregnancy, birth and the post-partum, offering health information, moral support, appointment reminders, and referrals to formal health care providers.

**Methods:**

An ethnographic study of the CommCare intervention and the larger maternal health program into which it fits was conducted in order to identify key social and cultural contextual factors that contribute to the uptake and functioning of this mHealth intervention in Senegal. Ethnographic methods and semi-structured interviews were used with participants drawn from four categories: NGO field staff (*n* = 16), trained health care providers (including physicians, nurses, and midwives) (*n* = 19), community level health care providers (*n* = 13); and women belonging to a community intervention known as the Care Group (*n* = 14). Data were analyzed using interpretive analysis informed by critical medical anthropology theory.

**Results:**

The study identified five socio-cultural factors that work in concert to encourage the uptake and use of CommCare: convening women in the community Care Group; a cultural mechanism for enabling pregnancy disclosure; constituting authoritative knowledge amongst women; harnessing the roles of older women; and adding value to community health worker roles. We argue that, while CommCare is a powerful tool of information, clinical support, surveillance, and data collection, it is also a social technology that connects and motivates people, transforming relationships in ways that can optimize its potential to improve maternal health care.

**Conclusions:**

In Senegal, mHealth has the potential not only to bridge the gaps of distance and expertise, but to engage local people productively in the goal of enhancing maternal health care. Successful mHealth interventions do not work as ‘magic bullets’ but are part of ‘assemblages’ – people and things that are brought together to accomplish particular goals. Attention to the social and cultural elements of the global health assemblage within which CommCare functions is critically important to understand and develop this mHealth technology to its full potential.

## Plain English summary

This paper reports on the results of a qualitative study of an innovative mobile health (mHealth) intervention to improve maternal health care in rural and remote areas of Senegal. CommCare is an application that runs on cell phones distributed to community level health workers known as *matrones*. Women enrolled in the system are tracked throughout pregnancy, delivery and the postpartum including postnatal check-ups and infant vaccinations. With the help of the device, *matrones* offer health information, moral support, appointment reminders, and can also make referrals directly to a Nurse or Midwife.

While mHealth technologies are emerging as part of the solution to many pressing global health issues, little is known about what makes them successful or not. Evidence is particularly wanting in terms of how mhealth interventions are understood and received at the local level. Social science scholarship demonstrates that technology adoption and use is complex and often unpredictable: context matters. In this study we sought to explore the socio-cultural factors that contributed to the uptake and functioning of CommCare and identified five key interconnected socio-cultural elements including: convening women in an intervention called the Care Group, a cultural mechanism for enabling pregnancy disclosure, constituting authoritative knowledge amongst women, harnessing the roles of older women, and adding value to the roles of community level actors. We argue that CommCare, while a powerful tool of information, surveillance, and data collection, is also a social technology that connects and motivates people and transforms relationships in ways that optimize the potential of the technology.

## Background

Although considerable progress has been made in reducing maternal mortality over the past 25 years in Senegal, the national maternal mortality ratio (MMR) sits at 315 per 100,000 live births [1]. When the data are disaggregated by both region and cause of death, an even more challenging picture emerges. Government statistics from 2013 showed that while the MMR was 389 in urban areas, it was 459 in rural areas overall, and as high as 700 in the most remote, underserviced areas of the country [2, 3]. Direct obstetrical causes (hypertensive disorders of pregnancy, haemorrhage, infection, unsafe abortion and obstructed labour) account for the majority of maternal deaths globally with the remaining due to indirect causes such as malaria and anaemia [4, 5]. The underlying causes of maternal morbidity and death in Senegal are much the same as they are for other low resource countries in Africa: insufficient access to health care during pregnancy and birth, lack of skilled health personnel to provide quality antenatal and delivery care, inefficient referral systems to care for women in obstetric emergencies, and the diminished socio-economic situations of many families that leave women in poor health. Many of these factors are exacerbated for women living in rural areas far from health facilities [6, 7].

The reduction of maternal mortality is a priority of Senegal’s Ministry of Health and Social Action (MOHSA) and mobile health (mHealth) technologies are emerging as part of the national approach [8]. Indeed, mHealth interventions such as rapid diagnostic tests, cell phone apps, and telemedicine, are changing the reality of health care delivery in low resource settings around the world [9]. Examples of mHealth interventions for maternal, neonatal and infant health include mobile phones used to send SMS reminders of antenatal care (ANC) appointments and encourage patient adherence and follow up, provide peer group support, support health workers in remote locations, provide health promotion information, facilitate referrals, collect clinical data and deliver test results [10]. It is worth noting that mobile phone penetration in Senegal, including rural areas, is high [11]; the vast majority of people either own or have access to a mobile phone and the wifi coverage is extensive. The majority of mHealth interventions for maternal health in low resource settings have been small-scale, donor-funded initiatives [12]. While they meet the demand within global health advocacy and funding circles to be innovative, they are not typically evidence based. Little is known about what makes them successful or not in terms of implementation and uptake. Evidence is particularly wanting in terms of how mHealth interventions are understood and received at the local level [13]. Social science scholarship demonstrates that technology adoption and use is complex and often unpredictable in any setting; context matters [14–16]. Therefore, research that can illuminate the context of technologies introduced in the name of global health ought to be part of the multi-disciplinary evidentiary mix.

In Senegal, mHealth has the potential not only to bridge the gaps of distance and expertise, but to engage local people productively in the project of improving maternal health. In rural and remote areas of the country, people rely mostly on health posts for primary care; health centres and hospitals with resident physicians are few and far between. Health posts are staffed by state-trained and employed nurses -- often men -- known as *Infermièr(e)s Chef de Poste* (ICP) and by midwives (*Sage-femmes*) -- usually women. They are assisted in their task of delivering primary health care to their vast constituencies by a cadre of community health workers, including *Agents de Santé Communautaires (*ASC) (community health workers) who offer limited primary care in community based structures know as *Cases de Santé* (health huts), and *matrones*, (community level birth attendants). Both types of community level health workers, while acknowledged by the state as essential, are volunteers trained and supported largely by non-governmental organisations.

Africare is a non-governmental organisation whose mission since its founding in the 1970s has been “Improving Lives and Building Futures” across Africa (https://www.africare.org/country/senegal/). In Senegal, Africare delivers programs in economic development, community health, and food security that support state infrastructures and policies particularly in rural and remote areas. Africare’s overall goal in the realm of maternal health has been to increase demand for quality health services, support locally driven behaviour change, and bolster the functioning of the state health system and its trained personnel by training and supporting community level health workers.

In 2014 Africare launched CommCare, an mHealth application that runs on cell phones distributed to *matrones* (or in some cases community health workers). It features health information and a service platform using text, pictures and voice in four languages, depending on the region. Building upon the community level interventions put in place during two USAID funded community health projects (*Projet Santé Communautaire* I (PSCI, 2008–2012) and II (2012–2016), CommCare is part of a project called *Collaborative Technologie à base communautaire pour ameliorer la Santé Maternelle et infantile au Sénégal* (CCHT) (Collaborative Community-based Technology to Improve Maternal and Child Health in Senegal) funded by Grand Challenges Canada in their Saving Lives at Birth (SLAB) stream. The basic application was created by Dimagi – a mobile health technology company – and tailored for use by Africare for the communities in which they work. Africare collaborated on the design of the CommCare platform with intensive input from community level and skilled health personnel.

The way that CommCare works is that *matrones* register pregnant women on the application, inputting all the pertinent health and contact information about the patient. The *matrone* follows the woman throughout her pregnancy, reminding her about the four recommended antenatal consultations, accompanying her in person to appointments, and supporting her in following medical advice, including tests, prescriptions and making a birth plan. CommCare also has an education and information component; the *matrone* is directed by the device to impart information about proper rest and nutrition during pregnancy and to inquire about danger signs and symptoms that might be cause for referral to the health post – such as swollen hands and feet or vaginal bleeding. The digital file for each woman generated by the *matrone* is automatically shared with the nurse or midwife at the local health post or health centre who also has a mobile phone with the application installed. A key feature of CommCare is the ability for *matrones* to make electronic referrals through the device directly to a nurse or midwife. In addition, CommCare inputs are uploaded in real time to the dashboard for monitoring and data collection at the district level.

Recent data generated by Africare demonstrates positive trends in terms of measurable increases in ANC attendance, deliveries with qualified personnel, and PNC attendance [17]. This study was conducted to learn more about why and how these improvements are working, focusing on the interconnectedness of the technology and its social and cultural context.

## Methods

This ethnographic study of Africare’s CommCare intervention and its larger maternal health program was conducted between January and June 2016 with follow up visits in May–June 2017 and May 2018. Ziguinchor we focussed on three regions: Tambacounda, Ziguinchor and Sedhiou. The first author, a medical anthropologist with training and extensive experience in qualitative methods, conducted the interviews and ethnographic observation while the second author contributed to study design and analysis. The qualitative methodology was intended to produce knowledge that would complement and augment data Africare collects routinely as part of its monitoring and evaluation practices. Ethnographic methods and semi-structured, open-ended interviews were used to look closely at what is often described in anthropological terms as ‘the particularities’ and ‘the pragmatics’ of CommCare on the ground. The interview schedule for each category of participant was designed to elicit information, experiences and personal reflections concerning the design and functioning of the CommCare intervention in the community. Field notes were also taken throughout. Evidence in ethnographic research is not confined to data generated by interviews but also includes the observations of the researcher in the field. For example, the researchers learned about Care Groups and the power dynamics within them not only from having them described in formal interviews, but by attending them. The total number of field visits and interviews ensured that data saturation – understood as a process, rather than a single point [18] – was achieved, ensuring the quality and reliability of the data. Triangulation is an inherent feature of ethnographic research in that data are drawn from a range of sources and by a range of methods – interviews, focus groups, field description, and documents – thereby increasing the validity of the research results [19, 20]. Recognition of the value of ethnographic methods in global health research has grown over the past decade [21–24]. While generated at the level of the particular, knowledge produced by ethnographic methods can provide insights that can contribute to national and global level health policy and practice.

A total of 62 interviews were conducted with participants drawn from four categories: Africare executive, technical and field staff coded as NGO (*n* = 16); state trained health care providers including physicians, nurses, and midwives coded as HCP (*n* = 19); community level health care providers including *matrones* and community health workers coded as MAT (*n* = 14); and women belonging to a community intervention known as the Care Group coded as CG (*n* = 13). The number of participants in Care Group interviews varied depending on the community, with the smallest being 6 and the largest more than 25. Interviews and Care Group group meetings took place with aid of translators and were occasionally attended in full or in part by other members of the community, such as representatives of the community health committee. Individual meetings with Africare staff and state trained formal health care providers took place in French with the first author alone. Interviews were typically conducted over the course of a single day field visit to each community in order to capture as many of the desired participants as possible from the target categories. The range in the number of participants from Care Group to Care Group was due to differing populations in communities and the availability of individuals on a given day. Interviews were recorded with permission of the participants and translated and transcribed into French.

The purpose of the study was to observe and describe the logic and structures of Africare’s maternal health interventions in Senegal, hear directly from Africare personnel who implement the programs in the field, talk to health professionals who deliver clinical and public health services that intersect with Africare programs and to hear from members of communities who are the targets of interventions about their motivations and experiences. Data analysis involved careful reading and coding of the transcripts and field notes in order ensure familiarity with the data. Codes were developed in relation to the broad objectives of the research and refined inductively in the initial reading of the data by the first author while applying interpretive anthropological analysis [25–27] informed by critical medical anthropology theory [28, 29]. For example, the initial code “Care Group” was refined into several sub-codes including “early pregnancy disclosure within Care Group” because the analysis of informants’ statements and field observations indicated the critical importance of the Care Group in facilitating early pregnancy disclosure. Saturation was achieved as broad themes emerged from the coding process followed by deeper nuance and insights related to those codes. Preliminary analysis was validated in meetings between the first and second author. Ethnography as a method provides opportunities for informal participant feedback throughout the research which contributes to the validation of preliminary findings and alerts the researcher to unexplored themes. Preliminary findings were also presented and validated at a series of participant feedback meetings in the health districts of Tambacounda and Ziguinchor and with Africare personnel in Dakar, a process intended to increase the “trustworthiness” and applicability of the research findings [30]. Five key elements were identified that together shape the use of CommCare in the context of Africare’s maternal health work in Senegal.

## Results

The main finding of this study is that successful interventions do not work as ‘magic bullets’ – even if a novel and powerful technology is deployed -- but rather function within assemblages. By assemblage we mean a set of elements, ideas, and actors that are brought together deliberately or spontaneously to accomplish a particular goal [31, 32]. While one aspect of an assembled global health intervention may be the most visible – as is the case with CommCare -- attention to how all the elements work together is critically important to understand and develop the intervention to its full potential. The key features of the CommCare assemblage include: convening women, enabling pregnancy disclosure, constituting authoritative knowledge, harnessing the roles of older women, and adding value to community level health workers. Access to electricity, phone credit, and the wifi network in rural areas where the project runs is also essential and Africare is working on its own innovative solutions in those area -- including partnerships with telecom companies and providing solar energy charging stations to health posts -- but in this research we focus on the social and cultural elements of the assemblage which tend to be under studied in global health research. Describing these elements in turn will illuminate our central argument that CommCare, while a powerful tool of information, clinical support, surveillance and data collection, is also a social technology that connects and motivates people and transforms relationships in ways that optimize the potential of the technology.

### Convening women: the Care Group

*Everything we do in maternal health here is carried by the Care Group. It is the Care Group that allows us to move things forward (NGO 8)*

A main vehicle through which Africare delivers its work to improve maternal health in Senegal is known as the Care Group. Care Groups consist of up to 25 women in a community who meet weekly and subscribe to the group with a small fee given each week into a communal pot. The Care Group draws on the tradition of women’s associations in Senegalese society and has therefore been well received. Care Group membership is voluntary and the percentage of women from each community who belong to a Care Group is variable. But when one Care Group begins to function well in a community, others have formed spontaneously and have reached out to Africare field staff for training and support. The purpose of the Care Group is twofold. First, it functions as a place where women receive information about the benefits of skilled care during pregnancy, birth and the post-partum period, and are encouraged to seek out prenatal care, make a birth plan, and deliver at a health facility with a skilled attendant posted by the MOHSA -- either a nurse with midwifery skills or a midwife. Second, the Care Group has a savings and lending function so that women can afford both the routine and unexpected costs of antenatal care and birth – medications, diagnostic tests, and transport -- a reality in the context of a health system that charges user fees.

*We contribute money to the Care Group every Saturday, and it is also the occasion when we discuss our health problems. Whenever a woman has a health problem she can count on the group to give her enough money for her care and then she can pay it back. (CG 2, 2016)*

*I think that since the setting up of the Care Group women come earlier for their prenatal consultations and then follow them. Children are vaccinated, women are better informed, and women receive support and guidance from marraines* (godmothers) *(CG 1, 2016)*

Care groups are accepted in the communities. Village chiefs, to whom we made courtesy visits as a matter of protocol before beginning our research for the day, were generally positive towards the intervention because they supported the goal of improving women’s health and noted the practical value of women saving money collectively for health care.

Nurses and Midwives posted to rural areas also observe the value of the Care Groups.

*The Care Group is an organization that aims to unite women for better management of their health. Since their installation, we have seen results. Honestly speaking, there have been results because I always say that it is not because they do not want to go to the hospital but because they do not know the dangers that lie ahead. So with the Care Group this allowed them to accentuate -- to multiply the talks, to multiply the home visits. At least every month they have four meetings. So every week, a meeting. And with their means of contribution that they make, the 50 Francs subscription, the money is there. So there is no “my husband has no money” problem. So that solved that problem. (HCP 19)*

The Care Group provides the social platform and enabling structure for other things to happen that are key to the success of the CommCare intervention. Several features of the Care Group, discussed below, facilitate the uptake and optimal use of CommCare because of the way they incorporate cultural norms.

### Enabling pregnancy disclosure

For CommCare to work, women must disclose their pregnancies and be registered. This simple truth illustrates the central role of the Care Group in encouraging women to disclose their pregnancies early and start antenatal care in a timely manner. Cultural norms in rural Senegal have women keeping their pregnancy status private for as long as possible. Women are concerned that a pregnancy disclosed too early is at risk of attracting *mauvais esprits* (bad spirits) and negative attention from others, which they identify as risks to themselves and their fetus. They prefer to wait until they begin to physically show – often well into the second trimester – which runs counter to best practices for antenatal care to begin in the first trimester and complete four antenatal consultations. Delaying disclosure presents a paradox for women who, as they become informed about the benefits of prenatal care through Africare’s education and information activities, find themselves in the difficult position of having to weigh the risks they perceive in early disclosure against the obstetrical risks of delayed antenatal care.

The power of the Care Group is that it works with, not against, the desire of women to keep their pregnancies private from the wider community well into the second trimester. During the Care Group meeting, a woman can signal discreetly to the others in the group that she is pregnant, often without even saying so aloud. Interviews with Africare technical and field staff alerted us to the practice which was confirmed during multiple field observations of Care Group meetings and Care Group interviews.

*Within the Care Group, women have a code. If someone falls pregnant, she wears a single earring and everyone knows that she is expecting. One doesn’t have to say anything. It’s the code of the group and no-one but the groups is aware. (NGO 8, 2016)*

*Before the formation of the Care Group, to identify a pregnant woman was a real problem. A woman would hide her pregnancy. But when we put in place the Care Group, in effect, we put in place a method for detecting pregnancy. So, when you come to a meeting, there is a secret code that says, see, this woman is pregnant. And like that, the Care Group members will know (CG 11 2017).*

The pregnant woman is then matched with a *marraine* from the group who becomes her guide and support person throughout her pregnancy, accompanying her to the community level health birth attendant – the *matrone* *–* as well as to antenatal consultations with the midwife or nurse at the health post. This creative use of the Care Group allows women to both comfortably disclose their pregnancies to the small group and discreetly begin pregnancy care, while at the same time keeping it secret from the larger community until a later date. This mechanism for enabling early pregnancy disclosure within the Care Group facilitates CommCare registration which, in turn, supports the course of clinical care.

*Before, the midwife had a hard time identifying women who needed prenatal care. But with registration in CommCare, each time a woman falls pregnant the Care Group directs her to go for her ANC visits. And when she is registered, it makes following her care a lot easier and they get better care. Truly, this CommCare has improved the situation of pregnant women and also their health during their reproductive years generally (CG 7, 2017)*

### Harnessing the authority of older women

Another way that the Care Group works with cultural norms, is by harnessing the power of older women, rather than seeing them largely as the perpetuators of harmful traditional knowledge and practices. Older women in Senegalese society (grandmothers and mothers in law) are sometimes the source of strain for pregnant women trying to navigate the new information and behaviour change expected of them in global health projects. As elders, they also tend to have more personal authority vis a vis younger men and women, especially their sons and daughters in law. “The husband has power, but mothers in law do too!” » (HCP 7, 2016).

In another subtle manoeuvre, the Care Group acknowledges the authority of older women and includes and transforms the traditional roles of older women in the community: grandmothers, (*belle-meres)* become marraines, and some former traditional birth attendants become *matrones,* rather than making them redundant and setting them up as “barriers” to progress around which daughters and daughters in law are expected to awkwardly (and impossibly) sidestep.

*In the Care Group we have women of reproductive age, pregnant women, breastfeeding women and we have grandmothers and this is a layer that should not be overlooked because they are the ones who often make the decisions about maternal health and having them in the Care Group also allows us to educate them. (NGO 7, 2016).*

In one Care Group interview, I asked a young mother to describe her recent experience with maternity care and childbirth. She told a brief story about her healthy pregnancy and delivery at the health post in which we were assembled. When the new mother fell quiet, the midwife, who had ambled over to join the meeting, cut in:

*There is the mother in law right there! She is the one who belongs to the Care Group. The daughter in law is new to the village and this is her first child. It was the mother in law sitting right there who went to her house and asked her: Are you following your antenatal appointments? No. She had done one and two but not three and four. So the mother in law brought the matrone to make the appointment and they went to the health post (CG 13, 2018)*

The other women in the group were nodding in agreement. The midwife went on:

*This lady, she is my friend in this work. Truly. Because she is really engaged. She is available. She accompanies the younger women. She is like the mother in law to all the women in this village. The others too (looking around) but at almost every delivery, she is there. (CG 13, 2018)*

Understanding and working with cultural norms around gendered and age related authority facilitates and strengthens the entire maternal health assemblage: it facilitates the uptake of health education and information and encourages early disclosure, which facilitates early enrolment in CommCare. Indeed, the viability of CommCare depends upon these elements.

### Constituting authoritative knowledge

When the Care Group is functioning well and is accepted by the larger community, it becomes a source of “authoritative knowledge” - a social science concept that is defined as “knowledge that gets to count” [33, 34]. Authoritative knowledge is knowledge upon which vital decisions can be made, such as the dispensing of money to buy medication for a pregnant woman, or the decision to transport her to a facility to give birth. Respect for the Care Group’s authority by the community as a whole marks a shift in the decision-making power in the community regarding pregnancy and birth towards *women as a group*. This aspect of the Care Group works with the reality that reproduction is not a solo endeavour in Senegal. (Nor is reproduction a solo endeavour in the West, but this characterization of women’s embeddedness in the power of family tends to be exaggerated for women in the global south). Senegalese women do not always have a great deal of individual decision making power, but rather they belong to families and communities in which husbands and belle-meres have a great deal of say over them. The Care Group generates collective power and authority, rather than asking individual women to stand up against community norms, expectations or constraints. The collective authority of the Care Group does not preclude the expression of individual ‘rights’ or empowerment – concepts at the centre of global health ‘reproductive and sexual health and rights’ frameworks -- but it does not demand it. It does not assume that women’s empowerment must be individualized to be real or to be effective in reaching the end goal: maternal survival and health. The Care Group allows women to develop social and financial solidarity.

*In the communities where we work, even if men appear to have the decision making power, women are the foundation … Care Groups educate and support not only women. I have seen Care Groups in which men are involved. I have attended community meetings in which men stand up and say that it is because of the Care Group that they now understand so much more about childbirth, and who credit care groups with helping not just pregnant women but the entire community (NGO 9, 2016)*

The testimony of the head of the community health committee who was present at a CG meeting bears this out.

*This project has allowed our mothers, our women and the ICP and his team to work well together. And as much as there have been difficulties in the past -- before this intervention many women hid their pregnancies -- today, as we speak this no longer happens, because in these women's groups, they help each other to push the woman who is able to go to the post to try to eliminate the complications in the pregnancies. Because if you do not go for prenatal visits there are many complications that can happen. I first learned about all this from my brother who started talking about it. At times I did not understand what the Care Group was. I would hear about it and I looked at it like one would a TV [from the outside]. But after these explanations I found it extremely important (CG 12, 2018).*

Reflecting on the subtle changes seen over time with regard to women’s empowerment in the communities in which they work, this respondent from Africare said

*What does the app bring in terms of the confidence of women in themselves, the power unleashed to women? You see, that is quite interesting. Because when I look at, when I visit [the region of] Maka, today, when I compare it to my first visit, I see that this is really something that has been brought out in women. They are more confident. They are more organized and engaged (NGO 11, 2018)*

Overall we contend that the authority of the Care Group subtly shifts power away from the sources of authority that hamper maternal health -- whether it is controlling treatment by belle-meres, the withholding of funds and decision making power by fathers and husbands, or misinformation that circulates in the community – to women as a group. Understanding the power of the Care Group in terms of its ability to build social and financial solidarity, to work with local cultural norms, to increase women’s confidence in managing their reproductive health affairs, and thereby to generate respect and authoritative knowledge is key to understanding CommCare as a social technology. Understanding and supporting these features optimizes its potential.

### Adding value to community health workers

CommCare was designed to be used by *matrones* – community level health care providers responsible for maternal and newborn health. Although skilled attendance in a functioning health facility remains a key goal of the global maternal health policy of the MOHSA in Senegal to improve maternal survival, the fact remains that many women – more than 50% in some regions of Senegal – continue to give birth in their communities [3]. Africare has been training and supporting *matrones* as a core feature of their maternal health programming for many years before the start of the CommCare project. The value of training of traditional birth attendants to contribute to maternal health has a been a matter of significant scientific debate. Training programs have been criticised for their brevity, poor quality, lack of follow-up, failure to account for cultural context, and lack of integration with the formal health care system [35–39]. Training programs for *matrones* in Senegal seek to avoid some of these pitfalls. *Matrones* train for up to 1 year (depending on previous experience) in an empirical, apprenticeship style with midwives and nurses, often living in Health Centres and Posts for the duration. In one of the first field visits during the research we were invited into the living quarters of the resident midwives, adjacent to the busy health post in which they worked. The midwives explained that the matrones had lodged with them for the duration of their training becoming close and growing to understand and respect each other, despite differences of socio economic background, education and language (Field Notes Feb 17, 2016). In these ways, matrones become strongly linked to the formal health care system; they are not outsiders. Furthermore, matrones reduce and ease the work burdens of nurses and midwives in ways they appreciate: they track women’s prenatal appointments, accompany women to the facility, provide language translation, offer continuous companionship during labour and the postpartum, often clean up after deliveries and fill in when the provider is absent or the delivery precipitous.

This matrone reflects on her work, noting both challenges and positive changes:

*With the training, one must practice, one must make an effort. But there have been many changes. There are women who miss their appointments. Sometimes the network isn’t working. But you have the example of the young woman sitting right there. She fell pregnant and we talked to her, we talked to her mother and she attended all four prenatal consultations and she gave birth under good conditions and has also followed the vaccinations (MAT 2, 2017)*

Health care providers in rural Senegal offered testimonies on the practical necessity of relying on matrones in rural areas. They praised their training and abilities with regard to identifying the signs of danger, making referrals correctly and even administrating some medications, such as misoprostol, at the community level, noting how CommCare had aided matrones’ abilities and motivation. « They are often at my side during births and they help with getting women to follow their prenatal and post natal appointments » (HCP 6, 2016).

One ICP recounted the following story when asked about how he viewed the work of matrones:

*When I was posted in [another town], there was a referral that went very well. The matrone sent me a message about a woman who was about to give birth with a posterior breech presentation. The message reached me just when I arrived in Bignona and when I saw it I came to the health post immediately and I met the ambulance and we sent the mother to Ziguinchor. [What’s important about the story] is that the referral was done properly, on time, and the matrone used the mobile to inform me. Otherwise it would have been a catastrophe (HCP 11, 2017)*

Another ICP with more than 20 years of experience, including many years at the most remote health post in the country, acknowledged that he could not do his work without matrones *“I have confidence in them” (HCP 16, 2018)* he stated. One challenge with the task shifting to matrones is that they continue to be engaged as volunteers who are trained and logistically supported, but not paid for their work. Most matrones are diligent and competent but we knew of several matrones who had lost their mobile phones or ‘given up’ their involvement in the CCHT project because of the need to work at other things.

CommCare adds value to the role of matrone in terms of her skills and social status; it strengthens the matrone’s social and affective ties to the women in her care; and it strengthens the matrone’s professional ties to nurses and midwives and increases the respect to which they are accorded. It is not the case that every matrone excels or every relationship is always positive, but what the observations in this study signal is the importance of the relationships of confidence and rapport. In these ways CommCare both relies on social relationships to function and contributes positively to the kinds of sociality and care that make good health care systems. As western biomedical systems are now realizing, the caring gap filled by auxiliary health workers is not a superficial extra, but an essential element of high quality care that contributes to good clinical outcomes. Recent scholarship identifies the crucial role of good client-provider communication and quality of care in both low and high resource settings [40–42].

This final quote below speaks to the positive attitude towards the value and capabilities of matrones, amongst skilled health care providers who work in rural and remote areas and a shift in perspective towards to seeing the task of improving maternity care increasingly in terms of public health education and community engagement, of which matrones are an important part.

*If the matrone is trained, she can do it. They give them the criteria: this woman you can care for; this woman you must refer. There is a chart on the wall of the Case and the device reminds them. And they do it correctly. It is community health, you see? It is a necessary evolution in a region such as this where access to a Health Post could be many kilometers away (HCP 17, 2018)*

The matrone is recognized by the Ministry of Health and Social Action in Senegal as having a role to play at the community level and as an adjunct to the clinical care of a skilled clinician; they are publically valorized once a year on the Day of the Community Health Worker [43, 44].

## Discussion

The idea of technology as social is well developed in medical anthropology and Science and Technology Studies (STS) but rarely have mainstream global health scholars engaged with this literature in order to understand how mHealth technology works (or not) on the ground. An excellent example of an STS approach to mHealth technology in a global health setting is a study by anthropologist Eileen Moyer on the use of cell phones to send peer to peer SMS messages to improve Antiretroviral Therapy (ART) adherence amongst HIV positive individuals in a poor neighbourhood in Nairobi, Kenya [45]. In her study, Moyer notes several aspects of the peer mentors’ work that mirrors our observations here about matrones. Peer mentors use the technology to fulfill a logistical function, she observes, but also to *care* about patients; these dual roles increase patient participation in treatment programs and the commitment of the peer to peer counselors. In contrast to our findings, however, Moyer notes that often formal health care workers do not know about or value the peer mentors’ technology-facilitated care and that this is, a weakness of the ART mHealth project. Thinking about technology as social is a novel approach in global health research and it can reveal important but hard to see aspects of how global health technologies work and the ways in which they can be supported.

Another relevent social science notion for understanding the social context of technology is authoritative knowledge. A central concept in a branch of medical anthropology known as the anthropology of reproduction, authoritative knowledge was first developed as a way to understand heirarchies in knowledge about birth and why some knowledge is privileged over other knowledge and becomes the basis on which critical decisions about care are made [33, 34, 46]. Using this idea, we identified subtle shifts in collective power and authority of Senegalese women who belong to Care Groups, vis-à-vis community norms and gender dynamics. The notion of authoritative knowledge reminds us that authority is not distributed in a dichotomous way beween men (who have power) and women (who do not) but is more subtle and mutable. Women’s authority in the global south need not look exactly like it does in the global north for us to acknowledge and work with it to further the ends of reducing maternal deaths.

Relatedly, the findings of this study contribute to an under-researched area within an otherwise extensive literature exploring women’s unequal decision-making power in the management of their own maternal health: the influence of mothers and mothers-in-law. These empirical observations counter dichotomous models of gender relations common in development literature. Recognizing and working with the powerful influence of older women in families and communities means that interventions to improve the use of ANC and increase the number of births with skilled attendants should not only target women of childbearing age and their husbands, but also, where relevant, older women [47, 48].

Finally, these findings are relevant to long standing inter-disciplinary debates on the role of traditional birth attendants in global maternal health [49–52]. Recent scholarship has re-ignited the question of whether and how TBAs can play a role in reducing maternal mortality, arguing that given the practical necessity of community level birth attendance in many places, their potential should be properly evaluated and their participation tailored to the particularities of the local context, so that trained community level maternity care workers can be part of the solution instead of part of the problem [53, 54]. This observation finds support within global level policy and research on local interventions for task shifting and task sharing defined as.

“the rational redistribution of tasks among health workforce teams [in which] specific tasks are moved, where appropriate, from highly qualified health workers to health workers with shorter training and fewer qualifications in order to make more efficient use of the available human resources for health” [55].

In addition, the emergence of a range of technical and pharmaceutical interventions proposed for use by TBA-like community actors (in some cases appropriately described as auxiliary midwives) is a major factor in revisiting and re-thinking the policies that have sidelined them. New clinical research is emerging to show that matrones in Senegal, for example, are capable of correctly managing PPH with misoprostol [56–58]. Such studies can trickle *up* to the national and global policy level [59].

Taken together, our findings signal a shift in how to think about and navigate the social and cultural context of mHealth interventions in global health settings. First, understand the particulars of a given setting rather than rely on broad generalisations about ‘other cultures’. Second, work with social and cultural norms rather than conceiving of them as barriers to knock down or overcome. Third, consider qualitative methodologies to illuminate the subtle aspects of the interconnectedness of technology and people.

There are a number of limitations of the study. First, participants were recruited because they were already involved in Africare’s community health activities and, therefore, may have been less likely to share highly critical opinions or experiences. Second, focus group interviews can privilege some voices over others. Yet this very limitation yielded insight into an important dynamic – the powerful role that older women play in the lives of their sons, daughters, and daughters in law. To mitigate against this, three small focus groups were conducted for young married women only. (All were older than 18). A final limitation of the study is that, given the need for translators to conduct some interviews or portions of interviews, some nuance may have been lost in the moment, though we did have the benefit of reading the transcripts later.

## Conclusion

CommCare is an mHealth innovation that has captured the imagination of local people and engaged a range of actors in the project of improving maternal and infant health in Senegal. It may seem an obvious point to make that technology is not autonomous, that it is, quite literally, in the hands of users who are part of communities and cultures. Although CommCare is the most visible, most attention grabbing feature of Africare’s efforts to improve maternal health in Senegal, it is a social technology, one part of an assemblage that is grounded by the Care Group and relies on the work of matrones and the skills and social relationships they develop in their communities and with formal health care providers. CommCare -- while a powerful tool of information, decision making support, recording keeping, accompaniment, and data collection – also connects and motivates people and transform relationships.

There was a diversity of vantage points across respondent groups. Though some midwives, nurses and Africare staff who worked closely with communities expressed frustration from time to time about matrones who lost cell phones or failed to learn how to use them quickly, and though some matrones and Care Groups complained they needed more support, all were in broad agreement about the value and potential of the CommCare intervention. There was also broad agreement across respondent groups about the socio-cultural factors that inhibited women’s uptake of ANC and assisted deliveries and therefore an appreciation of the ways that CommCare was socially embedded within the workings of the Care Group and matrone in a hands-on way.

The Ministry of Health and Social Action in Senegal (MOHSA) intends to roll out CommCare across the country and also add to its functionality with other features. Keeping the CommCare assemblage as we have described in this paper firmly in view may facilitate this scale up. But the findings from this study are not limited in relevance to Senegal nor to maternal health interventions. The authors of the Lancet Commission on Technologies for Global Health 2012 wrote: «Technology should be combined with other innovations, such as effective delivery mechanisms and novel approaches to financing if it is to be scaled-up and have a substantial effect on global health » [60]. Our findings accord with this observation and seek to add to it by naming and elaborating the range of novel and sometimes hard to see ‘innovations’ at the community level. Ethnographic and other modes of qualitative research that can illuminate the social and cultural context of technologies introduced in the name of global health ought to be part of the multi-disciplinary contributions to global health research and implementation.

## Data Availability

Data sharing is not applicable to this article as no datasets were generated or analysed during the current study.

## Abbreviations

ANC: Antenatal Care
CCHT: *Collaborative Technologie à base communautaire pour ameliorer la Santé Maternelle et infantile au Sénégal*
CHW: Community Health Worker
ICP: *Infermière Chef de Poste*
MOHSA: Ministry of Health and Social Action
NGO: Non-governmental organization
SLAB: Saving Lives at Birth
SF: *Sage Femme*
TBA: Traditional birth attendant
WHO: World Health Organisation
